# The Determinants of Non-compliance on Rabies Vaccination in North-West Peninsular Malaysia

**DOI:** 10.1007/s44197-022-00080-2

**Published:** 2023-01-09

**Authors:** Mohammad Fazrul Mohammad Basir, Suhaily Mohd Hairon, Tengku Alina Tengku Ismail, Che Muzaini Che’ Muda, Hamizar Iqbal Abdul Halim

**Affiliations:** 1 Department of Community Medicine, School of Medical Sciences, Health Campus, Universiti Sains Malaysia, 16150 Kubang Kerian, Kelantan Malaysia; 2 Communicable Disease Control Unit, Perlis State Health Department, Ministry of Health Malaysia, 01000 Kangar, Perlis Malaysia; 3 Kangar District Health Office, Ministry of Health Malaysia, 01000 Kangar, Perlis Malaysia

**Keywords:** Rabies, Vaccination, Non-compliance, Dog bite, Post-exposure prophylaxis

## Abstract

**Introduction:**

Rabies post-exposure vaccination (Rabies PEV) remains the most fundamental prevention of human Rabies if administered in a timely and appropriate manner. The study was aimed to determine the proportion and determinants of non-compliance on Rabies PEV among dog bite patients in Perlis, Malaysia from July 2015 to June 2020.

**Methods:**

A retrospective cohort study was conducted using Perlis Rabid Potential Animal Bite Registry data. Independent variables and compliance status were obtained from the registry. Logistic regression analysis was utilized on 507 dog bite patients.

**Results:**

Most of dog bite patients were age group of 46–60 years old (23.1%), male (61.3%), Chinese (49.5%), seeking treatment less than 24 h after the exposure (78.3%), category two of exposure (76.3%) and bitten on lower extremities (57.8%) by an owned dog (58.4%). Only 19.5% were non-compliance to Rabies PEV. Siamese had significantly two-timed (AOR: 2.00; 95% CI 1.06, 3.76) odd higher to become non-compliance. Being bitten during 3rd (AOR: 0.27; 95% CI 0.12, 0.59), 4th (AOR: 0.24; 95% CI 0.11, 0.52) and 5th (AOR: 0.20; 95% CI 0.09, 0.44) year of the outbreak had significantly lower odds to non-comply with Rabies PEV.

**Conclusion:**

19.5% of dog bite patients still did not comply with the Rabies PEV series. Siamese would likely to non-comply whereas bitten on the 3rd, 4th and 5th years of outbreak less tendency to non-comply. Continuous health promotion to the public in the various languages despite outbreak status are ongoing to improve the perception of risk and benefit toward compliance of Rabies PEV.

## Introduction

Rabies is one of the most commonly overlooked vaccine-preventable zoonotic virus diseases. It is well known for its mortality, with over 100% of instances resulting in death once the symptom appears in a human. Both domestic and wild animals can be infected with rabies. Domestic dogs, on the other hand, are blamed for the lion’s share of dog-mediated human Rabies [1]. According to a study, canine Rabies caused approximately 59,000 (95% CI, 25,000,159,200) human Rabies deaths globally each year, with the Asia and Africa continents playing a large role in this estimate. Furthermore, worldwide economic costs of roughly 8.6 billion USD and 3.7 million Disability Adjusted Life Years (DALY) lost were documented [2].

Rabies has been known to occur in Malaysia since 1845, although data on its occurrence have only been collected since 1924 [3]. Rabies has only been identified in Peninsular Malaysia, and it is more widespread in the northern states of the peninsula. The most recent human case was recorded in 1998, while the most recent canine Rabies cases were reported in 1999. Malaysia proclaimed itself a rabies-free nation to the OIE in July 2013, in compliance with Article 8.10.2 of Chapter 8.10 of the Terrestrial Animal Health Code (2012) [4]. Malaysia lost its free status due to a recent outbreak in Peninsula Malaysia's northern regions in 2015 [5]. Perlis had the highest number of canine rabies cases in this outbreak (20 cases), followed by Kedah (18 cases) and Pulau Pinang (4 cases). Malaysia recorded 34 episodes of human Rabies, 32 of which were fatal [6].

WHO formed a United Against Rabies partnership with the World Organization for Animal Health (OIE), the Food and Agriculture Organization of the United Nations (FAO), and the Global Alliance for Rabies Control (GARC). They designed a framework to eliminate dog-mediated human Rabies by 2030, with a zero human death vision from dog-mediated Rabies [7, 8]. One of the global strategic aims is to make vaccines and technologies more effective by guaranteeing fair, affordable, and timely access to health care, medicines, and vaccinations [8]. Rabies Post-Exposure Vaccination (Rabies PEV) provided in a timely and proper manner has a very potent effect in preventing and escaping the disease [9]. The American Committee on Immunization Practices accepted Malaysia's standard method for administering Rabies PEV, specifically the Essen 4-dose schedule, which consisted of intramuscular injections over the deltoid area on days zero, three, seven, and fourteen [10, 11]. The global noncompliance rate of Rabies PEV varies from 22.0 to 55.6% [12–17]. Previous studies revealed that the most significant obstacle for completion of Rabies PEV was the cost incurred for the vaccine [12, 15, 18]. However, in Malaysia, the claimed barrier does not exist because the Rabies PEV is offered for free in public hospitals. Lack of compliance with Rabies PEV has been identified as a key cause to human Rabies transmission and the end of Rabies-free countries [19, 20]. Recognizing the determinants of noncompliance with Rabies PEV in this context thus offers a great chance for even more effective health education and enhanced vaccination service delivery.

Nonetheless, to the best of the researcher’s knowledge, there has been no published study on Rabies PEV in Malaysia. The current study sought to ascertain the proportion and determinants of non-compliance with Rabies PEV among dog bite patients in Perlis from July 2015 to June 2020. As a result, an understanding of the focused preventive plan is provided to optimize Rabies PEV compliance in the future.

## Methods

### Study Setting

Between July 2015 and June 2020, a retrospective cohort analysis was conducted on dog bite patients who sought treatment after exposure in any healthcare facility and were recorded in the Perlis Potential Rabid Animal Bite Registry. Perlis is bounded by Thailand's Satun and Songkhla Provinces, as well as Malaysia's state of Kedah. This state has a population of 254,700 people and covers an area of 819 kms square (km^2^) [21]. North-West Peninsular Malaysia includes state of Perlis, Kedah, Pulau Pinang and Perak. Based on study by [22], the number of canine Rabies were reported in Perlis was 20 canine Rabies whereas Kedah (18 cases) followed Pulau Pinang (4 cases). Thus, Perlis was chosen as study location as it had the most significant number of canine Rabies cases as compared to other states in North-West Peninsular Malaysia [22].

### Criteria for Sample Selection

The inclusion criteria for sample selection were that the patient had a history of dog bite exposure and had received at least one Verorab vaccination. However, if a participant reported more than one exposure during the research period, only the data corresponding to the initial exposure was included; subsequent exposure reports and Rabies PEV were excluded. Patients who resumed the vaccine series despite Perlis were not included in this research.

### Sample Size Determination and Sampling Method

The sample size was calculated using Power and Sample Size Calculation Software for single and two proportions with a type I error of 5% and a type II error of 20%. The calculated sample size was 486; after 10% for the data error consideration, the required sample size was 535 [23]. However, only 507 cases fulfilled the criteria for sample selection from 587 cases of rabid potential animal bites recorded in the Perlis Rabid Potential Animal Bite Registry. Thus, no sampling was applied for this study.

### Data Collection and Research Tools

This study used a surveillance database of the rabid potential animal bites which was in the form of a registry. No personal information was included in the proforma to maintain the anonymization of the data set. The data were reviewed to match the inclusion and exclusion criteria, cleaned, and explored.

#### Perlis Rabid Potential Animal Bite Registry

The Perlis State Health Department created this register in 2015, when the Canine Rabies outbreak occurred. It is in Microsoft Excel format and is kept at the Crisis Preparedness and Response Centre (CPRC) Perlis. The attending doctors notified Kangar Medical Officer of Health for all dog bite patients who visited any healthcare institution in Perlis. The dog bite cases were investigated by the Assistant Environmental Health Officer using the standard investigation form and faxed to CPRC Perlis. The information was then recorded into the Perlis Rabid Potential Animal Bite Registry. The registry includes independent factors such as sociodemographic data (age, sex, and ethnicity), animal factor (type of dog ownership), exposure factor (category of exposure, site of injury), and treatment factor (RIG recipient). The registry employed WHO definitions for the categories of exposure, which are as follows: (1) Category one of exposure is defined as touching or feeding an animal, licks on intact skin, (2) Category two exposure is described as bare skin nibbling or minor scratches or abrasions without bleeding and (3) Single or numerous transdermal bites or scratches, contamination of mucous membranes or broken skin with saliva from animal licks, and exposures due to direct contact with a dog are all classified as category three exposures [9]. In this study, the site of injury was divided into three categories: upper extremities (hands, arm, elbow, and fingers), lower extremities (leg, calf, foot, and toes), and head and body (injury on the head, face, neck, chest, back, abdomen, anogenital area). This study generated factors including age group, time elapses for treatment, years of exposure, and compliance level. The time elapsed for treatment was calculated using the date of exposure and the date of vaccination start, as documented in the registry. On July 1, 2015, Perlis had the first dog bite incidents caused by a rabid dog, and a Rabies outbreak was proclaimed on the same day. As for years of exposure, we consider the first year as 1st July 2015 to 30th June 2016, followed by the 5th year ending on 30th June 2020. Despite the fact that the Rabies PEV was only administered at the government hospital, the variable of type healthcare facilities utilized in this study was defined as the first healthcare facility visited by dog bite patients for treatment or evaluation following exposure. We divide facilities into two types: primary health clinics, which include both government and private health clinics, and hospitals, which include both government and private hospitals.

On a weekly basis, the Medical Division of the Perlis State Health Department gathered the actual date to hospital for the vaccine, including both the first and follow-up vaccination series, and recorded it into the Perlis Rapid Potential Animal Bite Registry. The date in the register is utilized to establish the dependent variable status: compliance or non-compliance with the Rabies PEV. Non-compliance is defined as the dog bite patients who discontinued being vaccinated at any stage throughout the prescribed regimen (except those who discontinued vaccination after three doses, where the dog remains healthy and alive for at least ten days after the exposure). Otherwise, compliance is defined as the dog bites patients who completed the recommended course, including those who got the vaccine beyond the scheduled date of vaccination.

### Statistical Analysis

The data were analyzed using SPSS version 26.0 software. Descriptive statistics were used to summarize the sociodemographic characteristics of subjects. Numerical data were presented as median (IQR) based on their normality distribution. The normality distribution was determined based on the finding of the histogram, Shapiro–Wilk test, skewness, and kurtosis. Frequency (percentage) was used to present the categorical data. The proportion of non-compliance on Rabies PEV was expressed in percentage. The nominator was the number of dog bites patients who are classified as non-compliance. The denominator was the number of dog bite patients registered in the Perlis Potential Rabid Animal Bite Registry between 1 July 2015 and 30 June 2020, whose fulfilled inclusion and exclusion criteria.

Multiple logistic regression was used for the analysis to determine the determinants of non-compliance. Simple logistic regression was performed first to select the preliminary variables that have an association with non-compliance and presented as Crude Odd Ratio (OR). Variables with *p* values less than 0.25 were selected for multiple logistic regression and the Backward Likelihood Ratio (LR) method was applied for this purpose. Collinearity and interaction between the significant variables as well as the Goodness of fit model were checked. The fitness of the model was tested using the Hosmer–Lemeshow goodness of fit test (*p* value > 0.05), the classification table (> 80%), and the receiver operating characteristic (ROC) curve (> 70%). The significance level was set at 0.05.

## Results

From July 2015, when the Canine Rabies outbreak began, through June 2020, there were 587 reports of individuals bitten by rabid potential animals to the Kangar District Health Office. However, 41 records (7.0%) of people bitten by animals other than dogs, 26 records (4.4%) of people receiving no Rabies PEV at all, seven records (1.2%) of people known to continue their vaccination in a location other than Perlis, and six records (1.0%) of people experiencing ‘subsequent exposure’ were excluded from the study out of 587 patients. A maximum of two recorded exposures were reported for an individual. Finally, 507 patients (86.4%) were enrolled in this study.

### Sociodemographic Characteristic of Dog Bite Patients

The median age of dog bite patients was 37.50 (41.00) years old, with the most common age group being 46 to 60 years old (23.1%). The majority of dog bite patients (61.3%) were male, Chinese (49.5%), and had been bitten by an owned dog (58.4%). The overall median (IQR) for the time elapse for Rabies PEV was 0.00 (30.00) days, with 78.3% seeking treatment within 24 h of exposure. In terms of exposure, the lower extremities is the most bitten site, accounting for 57.8% of all cases and predominating by category two of exposure (76.3%). The hospital (76.1%) is a first-choice healthcare facility for dog bite treatment. Table 1 displayed the comprehensive descriptive information for all of the samples included in the analysis.

**Table 1:** Descriptive sociodemographic, animal, exposure, and treatment factor of dog bite patients in Perlis between July 2015 and June 2020 (*n* = 507)

Variables	Total *n* (%)	Non-compliance *n* (%)	Compliance *n* (%)
Age (years old)^a,b^	37.50 (41.00)	33.00 (31.00)	43.00 (42.00)
0–15	116 (22.9)	23 (19.8)	93 (80.2)
16–30	82 (16.2)	19 (23.2)	63 (76.8)
31–45	84 (16.6)	23 (27.4)	61 (72.6)
46–60	117 (23.1)	19 (16.2)	98 (83.8)
> 60	107 (21.1)	15 (14.0)	92 (86.0)
Sex			
Female	196 (38.7)	33 (16.8)	163 (83.2)
Male	311 (61.3)	66 (21.2)	245 (78.8)
Ethnicity			
Others	116 (22.9)	31 (26.7)	85 (73.3)
Siamese	140 (27.6)	24 (17.1)	116 (82.9)
Chinese	251 (49.5)	44 (17.5)	207 (82.5)
Dog ownership status^b^			
Not owned	115 (22.7)	38 (33.0)	77 (67.0)
Owned	296 (58.4)	53 (17.9)	243 (82.1)
Unknown	95 (18.7)	8 (8.4)	87 (91.6)
Site of injury			
Lower extremity	293 (57.8)	51 (17.4)	242 (82.6)
Upper extremity	171 (33.7)	40 (23.4)	131 (76.6)
Head & body	43 (8.5)	8 (18.6)	35 (81.4)
Category of exposure			
III	90 (17.8)	18 (20.0)	72 (80.0)
II	387 (76.3)	71 (18.3)	316 (81.7)
I	30 (5.9)	10 (33.3)	20 (66.7)
Year of Exposure			
1st year of the outbreak	124 (24.5)	10 (8.1)	114 (91.9)
2nd year of the outbreak	78 (15.4)	4 (5.1)	74 (94.9)
3rd year of the outbreak	107 (21.1)	28 (26.2)	79 (73.8)
4th year of the outbreak	108 (21.3)	29 (26.9)	79 (73.1)
5th year of the outbreak	90 (17.8)	28 (31.1)	62 (68.9)
Time elapsed for Rabies PEV (days)^a^	0.00 (30.00)	0.00 (1.00)	0.00 (30.00)
< 1 day	397 (78.3)	74 (18.6)	323 (81.4)
1 day	72 (14.2)	19 (26.4)	53 (73.6)
≥ 2 days	38 (7.5)	6 (15.8)	32 (84.2)
RIG receipt			
Yes	25 (4.9)	6 (24.0)	19 (76.0)
No	482 (95.1)	93 (19.3)	389 (80.7)
Type of healthcare facility			
Primary health care	121 (23.9)	13 (10.7)	108 (89.3)
Hospital	386 (76.1)	86 (22.3)	300 (77.7)

^a^Median (IQR)

^b^Missing data of 0.2% (*n* = 506)

### Proportion of Non-compliance for Rabies PEV

The proportion of non-compliance for Rabies PEV in Perlis from July 2015 till June 2020 was 19.5% (95% CI 16.1, 23.0) consisting of 99 patients. The remaining 408 patients (80.5%) complied with the Rabies PEV series for the same period in Perlis.

### Determinants of Non-compliance on Rabies PEV

Simple logistic regression was performed and variables with a p value less than 0.25 were selected into multiple logistic regression. The final model of multiple logistic regression in our study demonstrates that Siamese has significantly two-timed (Adjusted OR (Adj. OR): 2.00, 95% CI 1.06,3.76, *p* value = 0.031) odd higher than other ethnicities when controlling the year of the outbreak. Dog bite patients bitten on the 3rd year of outbreak significantly has a 0.73 odd lower (Adj. OR = 0.27; 95% CI 0.12,0.59; *p* value = 0.001) whereas bitten on the 4th year of outbreak significantly has 0.76 lower odds (Adj. OR = 0.24; 95% CI 0.11,0.52; *p* value < 0.001) to become non-compliance compared to bite in the first years of the outbreak when adjusting for ethnicity. Patients who were bitten by a dog on the 5th year of outbreak significantly have 0.8 odds lower (Adj. OR = 0.20; 95% CI 0.09, 0.44; *p* value < 0.001) compared bitten on the first years of the outbreak receiving an incomplete course of Rabies PEV when controlling the ethnicity. Tables 2 and 3 illustrate the complete findings for the simple and multiple logistic regression in this study.

**Table 2:** Simple logistic regression of factors associated with non-compliance of Rabies PEV among dog bite patients in Perlis (*n* = 507)

Variables	*B*	Crude OR (95% CI)	Wald Stat. (*df*)	*p* value
Age (years old)				
0–15		1		
16–30	− 0.2	0.82 (0.41, 1.63)	0.32 (1)	0.571
31–45	− 0.42	0.66 (0.34, 1.27)	1.56 (1)	0.212
46–60	0.24	1.28 (0.65,2.49)	0.51 (1)	0.477
> 60	0.42	1.52 (0.75, 3.09)	1.32 (1)	0.251
Sex				
Female		1		
Male	− 0.29	0.75 (0.47, 1.19)	1.47 (1)	0.226
Ethnicity				
Others		1		
Siamese	0.57	1.76 (0.97, 3.22)	3.42 (1)	0.065
Chinese	0.54	1.72 (1.02, 2.90)	4.07 (1)	0.044
Dog ownership status				
Not owned		1		
Owned	0.82	2.26 (1.39, 3.69)	10.71 (1)	0.001
Unknown	1.68	5.37 (2.36, 12.21)	16.06 (1)	< 0.001
Site of injury				
Lower extremity		1		
Upper extremity	− 0.37	0.69 (0.43,1.10)	2.44 (1)	0.118
Head & body	− 0.08	0.92 (0.40,2.11)	0.04 (1)	0.847
Category of exposure				
III		1		
II	0.11	1.11 (0.20,1.25)	0.13 (1)	0.717
I	− 0.69	0.50 (0.63,1.98)	2.19 (1)	0.139
Year of exposure				
1st year of the outbreak		1		
2nd year of the outbreak	0.48	1.62 (0.49, 5.37)	0.63 (1)	0.427
3rd year of the outbreak	− 1.40	0.25 (0.11, 0.53)	12.41 (1)	< 0.001
4th year of the outbreak	− 1.43	0.24 (0.11, 0.52)	13.14 (1)	< 0.001
5th year of the outbreak	− 1.64	0.19 (0.09, 0.43)	16.72 (1)	< 0.001
Time elapsed for Rabies PEV (days)				
< 1 day		1		
1 day	− 0.45	0.64 (0.36, 1.14)	2.28 (− 1)	0.131
≥ 2 days	0.20	1.22 (0.49, 3.03)	0.19 (− 1)	0.665
RIG receipt				
Yes		1		
No	0.28	1.32 (0.51, 3.40)	0.33 (1)	0.564
Type of healthcare facility				
Primary health care		1		
Hospital	− 0.87	0.42 (0.23, 0.78)	7.45 (1)	0.006

**Table 3:** Multiple logistic regression of factors associated with non-compliance of Rabies PEV among dog bite patients in Perlis (*n* = 507)

Variable	*B*	Adjusted OR (95% CI)	Wald Statistic (*df*)	*p* value
Ethnicity				
Others		1		
Siamese	0.69	2.00 (1.06, 3.76)	4.64 (1)	0.031
Chinese	0.46	1.58 (0.91, 2.74)	2.63 (1)	0.105
Year of exposure				
1st year of the outbreak		1		
2nd year of the outbreak	0.59	1.80 (0.54, 6.00)	0.92 (1)	0.337
3rd year of the outbreak	− 1.32	0.27 (0.12, 0.59)	10.82 (1)	0.001
4th year of the outbreak	– 1.43	0.24 (0.11, 0.52)	13.00 (1)	< 0.001
5th year of the outbreak	− 1.63	0.20 (0.09, 0.44)	16.18 (1)	< 0.001

The backward method was applied

No multicollinearity and interaction

Hosmer Lemeshow test, *p* value: 0.397

Classification table correctly classified: 80.5%

The area under Receiver Operating Characteristic (ROC): 0.703

## Discussion

Regardless of the risk exposure of the suspected rabid animal bite especially dogs, the uttermost prevention strategy of human Rabies is early and proper administration of the Rabies PEV [9]. As in Malaysia, there were established guidelines by the Ministry of Health for appropriate management of Rabies PEV since 2015 whereby the Rabies PEV comprises 4 series of injections over 2 weeks [10]. However, there is still a barrier for the dog bite patients to complete the series.

This study found that between 2015 and 2020, children under the age of 15 made up just 22.9% of all dog bite cases in Perlis. This conclusion contradicted recent research that found that children and teenagers accounted for more than two-thirds of dog bites [24, 25]. Children are more vulnerable to dog bites due to their smaller size and provocative attitudes toward the dog. According to the Asian Rabies expert, this age group is less likely to disclose dog exposure to their parents, such as licking or slight scratching resulting in lower health-seeking behavior at the health care institution [26].

It is predicted that the majority of the samples will be exposed to category II, which is similar to previous research [16, 23, 27]. The recommended for the initiation of Rabies PEV is reserved for patients who have experienced category II or III exposure to a rabid potential dog [9, 10]. Surprisingly, 30 dog bite patients (5.9%) in the current study were classed as category I of exposure yet received the Rabies PEV. Two-thirds had finished the vaccination regimen. It is difficult to distinguish the cause for the Rabies PEV commencement, whether the patient requests the vaccine, or the practitioner prescribes the vaccination despite the lower risk of exposure. Similar outcomes were seen in research on physician evaluation and PEP prescription practise [28]. As a result, practitioners emphasizing the necessity of clinical exposure evaluation for dog bite patients results in effective exposure classification and management.

The COVID-19 pandemic has resulted in a significant loss of human life throughout the world, and it poses an unprecedented threat to public health, healthcare, and the workplace [29]. Despite that, the Rabies PEV was not affected by the pandemic COVID-19 as it is an equally essential service that should be provided in the government healthcare facility. Based on the descriptive data during the 5th year of the outbreak, there was a slight reduction in the number of dog bite patients and a slight increment in the percentage of non-compliance. This reduction could be due to a lack of outdoor activity during the lockdown period which exposes the public to a dog bite or fear of attending hospital due to the risk of contracting COVID-19 after getting bitten by the dog [30, 31].

The current study’s 19.5% noncompliance proportion was much lower when compared to past studies in Senegal (45.5%) and India (46.8%); however, when compared to Cambodia (7.8%), this study’s findings were higher [15, 17, 27]. In Senegal, the cost of a complete Rabies PEV course without RIG was around 60 Euro, or 37.5% of the average monthly disposable wage (160 Euro) based on the World Development Indicator for Senegal [15]. Rabies PEV is offered free of charge in government hospitals in Malaysia. Aside from that, in Cambodia, the Rabies PEV is subsidized rather than free. As a result, because they have already paid the entire fee upfront, it encourages the patients to complete the vaccination series [27].

There is a discrepancy in the Rabies PEV regime between India and Malaysia. They employed the Updated Thai Red Cross intradermal post-exposure rabies prophylaxis regimen, which is lengthier (across 28 days) and so had a greater risk of noncompliance [17]. In Malaysia, the standard protocol for administering Rabies PEV consists of four injections spaced out over 14 days [10]. Shortening the entire course of Rabies PEV's length will assist improve patients' compliance, minimizing the burden on patients in terms of lost work time and transportation expenditures [26, 32]. This hypothesis explains why Malaysia has a lower proportion of non-compliance as compared to India.

This study also shown that ethnicity was a determinant in noncompliance with Rabies PEV. When compared to other ethnicities, Siamese people are twice as likely to be non-compliant. According to the Malaysian Population and Housing Census of 2000, an estimated 60,000 Siamese ethnic people accounted for less than 1% of Malaysia's population [33]. This minority ethic may struggle to understand Rabies' health education materials, which are only available in Malays or English, resulting in poor health literacy. Previous research in North Carolina found evidence of low Rabies awareness among minorities as a result of similar issues [34]. Several of them hold Thai citizenship are also married to Malaysians. Previous research revealed that cultural beliefs impacted their perspective of health and treatment choices [35]. As a result, we anticipate that non-Malaysian citizenship Siamese ethnic may return to their homeland after having first treatment in Malaysia, resulting in non-compliance with the Rabies PEV series.

Furthermore, our study showed that years of exposure are associated to noncompliance with Rabies PEV. However, it is not one of the determinants that affect noncompliance with the Rabies PEV. When adjusted for ethnicity, dog bite patients bitten from 1st July 2017 to 30th June June 2018 (third year of the Perlis Rabies outbreak) were 73% less likely to become non-compliance than those bitten from 1st July 2015 to 30th June 2016, which was the first year since the Perlis Rabies outbreak was declared. Finally, the odds of receiving an incomplete Rabies PEV series were lower from 1st July 2018 to 30th June 2019, the fourth year of the Perlis Rabies outbreak (by 76%), and from 1st July 2019 to 30th June 2020, the fifth year of the Perlis Rabies outbreak (by 80%). Thus, our findings contradicted recent research conducted in Bhutan and Cambodia, which found that those bitten later in the study period were more likely to have an incomplete series of the Rabies PEV [13, 27].

The findings in this study might be explained by the fact that human Rabies cases and outbreaks in Sarawak, Malaysia were reported on July 1st, 2017. During the third year of the Perlis Rabies outbreak, 11 human Rabies cases were documented in Sarawak [36, 37]. Following that, eight and five human Rabies cases were reported in Sarawak’s Rabies outbreak during the fourth and fifth years of Perlis' Rabies outbreak [38, 39]. Sarawak is located on the island of Borneo, which is not part of Peninsular Malaysia. Malaysia (Sabah and Sarawak), Brunei, Kalimantan, and Indonesia make up the Borneo islands. Although Sarawak is not part of peninsular Malaysia, human Rabies is a national health concern that has received extensive coverage in the media and on social media. Over the last three years of data collection, the Director-General of Health Malaysia’s regular updates through mass and social media served as a wake-up call to Perlis residents about the necessity of Rabies PEV’s completion. As a result, it is likely explanation for why compliance improved in years 3, 4, and 5. Messages in the media are effective at raising awareness and increasing demand for the Rabies PEV [40, 41]. This mass media reportage, when applied in combination with the health belief model, enhances the perceived of vulnerability and severity of dog bite patients, resulting in positive changes in Rabies PEV compliance.

### Study Strength and Limitation

This study sought to be a comprehensive study that incorporates data provided by various institutions within the Perlis State Health Department on handling dog bite cases, which contributes the most to this study's strength. Data for the registry were acquired through primary care (government and private), hospitals, and field investigations. As a consequence, our study outperformed a previous study that was confined to a single institution [12, 15, 42]. Our findings are beneficial not only to the health department but also to other relevant authorities, especially those in the one health approach. For example, the Veterinary Service Department used our localized demographic of dog bite patients to strategize the target population priority for the dog bite preventions besides utilizing these findings to encourage the dog owners to vaccinate their pets.

The limitation of our study on the determinants of non-compliance with Rabies PEV is that we did not conduct an in-depth interview with patients to determine why they did not comply with the Rabies PEV as indicated. This compliance behavior is generally the result of complex social, economic, cultural, accessibility, and other interacting elements that a basic categorical descriptive database cannot reflect on, making it impossible to reflect on the causes of non-compliance with the Rabies PEV. This approach supplements the qualitative or mixed-method study design to provide a more comprehensive picture of the local context on noncompliance behavior, which is recommended in the future. Because this investigation was intended as a retrospective cohort study utilizing secondary data, there is a dearth of information in the registry on certain variables such as education status, citizenship, socioeconomic level, and type of exposure. To establish evidence on the risk factors, it is advised that this study be conducted in a prospective cohort. Ideally, this would allow healthcare practitioners to identify dog bite patients at the time of reporting who fulfill the determinants that place them at a higher risk of not completing the course, allowing for targeted assistance. Nonetheless, our research has persuaded policymakers that, even with services supplied at practically no cost, one-fifth of our population may take the Rabies PEV series for granted. Our findings on the determinants of noncompliance on Rabies PEV may be used as a slice of scientific evidence for future promotion planning to attain elimination status by 2030.

## Conclusions

Despite the vaccine being available, accessible, and affordable most of the time in Malaysia, a segment (19.5%) of the population in North-West Peninsular Malaysia did not comply with the Rabies PEV series, putting them at a higher risk of developing human Rabies. Dog bite patients of Siamese ethnicity are more likely to possess an incomplete Rabies PEV series, although those bitten in the third, fourth, and fifth years of the outbreak are less likely to become non-compliant. As a result, it is advised that health education materials be prepared in a variety of languages, including Thai. Continuous public advocacy of Rabies prevention by top Ministry of Health officials, regardless of outbreak status, is needed to boost the perception of risk and benefits toward Rabies PEV compliance.

## Data Availability

Data are available on reasonable request from Director General of Health Malaysia.

## Abbreviations

PEV: Post-exposure vaccination
OIE: World Organisation for Animal Health
CPRC: Crisis Preparedness and Response Centre
WHO: World Health Organization

## References

[1] Fooks AR, Jackson AC, (eds). Rabies scientific basis of the disease and its management, 4th edn. Academic Press; 2020.

[2] Hampson K, Coudeville L, Lembo T, Sambo M, Kieffer A, Attlan M (2015). Estimating the global burden of endemic canine rabies. PLoS Negl Trop Dis.

[3] Ganesan J, Sinniah M (1993). Occurrence of human rabies in Peninsular Malaysia. Med J Malaysia.

[4] Vallat B, (ed). Bulletin. vol. 4. World Organisation for Animal Health (OIE); 2013.

[5] Navanithakumar B, Sohayati AR, Rohaiza Y, A SD, Leonora TM, Dorothy KS. An overview of rabies outbreaks in Malaysia, Ordinances and Laws. Malays J Vet Res (Putrajaya) 2019;10:148–58. https://www.dvs.gov.my/dvs/resources/user_16/MJVR%20Vol.10%20No.2%20(2019)/MJVR-V10N2-p148-158.pdf. Accessed 20 Oct 2020

[6] Abdullah NH. Kenyataan Akhbar KPK 24 Mei 2021 – Situasi Terkini Rabies di Sarawak – From the Desk of the Director-General of Health Malaysia 2021. https://kpkesihatan.com/2021/05/24/kenyataan-akhbar-kpk-24-mei-2021-situasi-terkini-rabies-di-sarawak/ (accessed 28 May 2021).

[7] Global Alliance for Rabies Control. Report of the Rabies Global Conference. Geneva 2015.

[8] World Health Organization, Food and Agriculture Organization of the United Nations, World Organisation for Animal Health, Control GA for R. Zero by 30: the global strategic plan to end human deaths from dog-mediated rabies by 2030. Geneva: 2018.

[9] World Health Organization. WHO expert consultation on rabies: third report. World Health Organization; 2018.

[10] Ministry of Health Malaysia. Interim Guideline for Human Rabies Prevention & Control in Malaysia 2015:17.

[11] Rupprecht CE, Briggs D, Brown CM, Franka R, Katz SL, Kerr HD et al. Use of a Reduced (4-Dose) Vaccine Schedule for Postexposure Prophylaxis to Prevent Human Rabies. MMWR Recomm Rep 2010;59:1–12. https://www.cdc.gov/mmwr/pdf/rr/rr5902.pdf. 20300058

[12] Romero-sengson RF. Factors affecting compliance to rabies post-exposure prophylaxis among pediatric patients seen at the research institute for tropical medicine. PIDSP Journal 2013;14. http://pidsphil.org/pdf/Journal_12312013/jo45_ja01.pdf. Accessed 25 Aug 2020

[13] Tenzin, Dhand NK, Ward MP. Human rabies post exposure prophylaxis in Bhutan, 2005–2008: Trends and risk factors. Vaccine 2011;29:4094–101. 10.1016/j.vaccine.2011.03.106 10.1016/j.vaccine.2011.03.106 21497633

[14] Tran CH, Kligerman M, Andrecy LL, Etheart MD, Adrien P, Blanton JD (2018). Rabies vaccine initiation and adherence among animal-bite patients in Haiti, 2015. PLoS Negl Trop Dis.

[15] Diallo MK, Diallo AO, Dicko A, Richard V, Espié E (2019). Human rabies post exposure prophylaxis at the Pasteur Institute of Dakar, Senegal: Trends and risk factors. BMC Infect Dis.

[16] Tran CH, Afriyie DO, Pham TN, Otsu S, Urabe M, Dang AD (2019). Rabies post-exposure prophylaxis initiation and adherence among patients in Vietnam, 2014–2016. Vaccine.

[17] Vinay M, Mahendra BJ, Nagaraj Goud B, Asha B, Ananthachari KR, Sheethal MP, et al. Socio-demographic characteristics affecting compliance to intra dermal rabies vaccination at anti rabies clinic in a Government Tertiary Care Hospital in Karnataka. J Evol Med Dental Sci 2013;2:7090–5. http://www.jemds.com/data_pdf/3_Ragini.pdf. Accessed 25 Aug 2020

[18] Sambo MB. Epidemiological dynamics of rabies in Tanzania and its impacts on local communities [MSc Ecology and Environmental Biology thesis, University of Glasgow]. In: PQDT - UK & Ireland. 2012. http://theses.gla.ac.uk/id/eprint/3663

[19] Guo C, Li Y, Huai Y, Rao CY, Lai S, Mu D (2018). Exposure history, post-exposure prophylaxis use, and clinical characteristics of human rabies cases in China, 2006–2012. Sci Rep.

[20] Ghosh S, Rana MS, Islam MK, Chowdhury S, Haider N, Kafi MAH (2020). Trends and clinico-epidemiological features of human rabies cases in Bangladesh 2006–2018. Sci Rep.

[21] Department of Statistics Malaysia Official Portal 2021. https://www.dosm.gov.my/v1/index.php?r=column/cone&menu_id=UDZaUXd2N2k2L2orK2FpdDJ1UjVtZz09 (accessed 6 Mar 2022).

[22] Bamaiyi H (2015). 2015 Outbreak of canine rabies in Malaysia: review, analysis and perspectives. J Vet Adv.

[23] Bariya BR, Patel S v, Shringarpure KS. Comparison of compliance of animal bite patients to two different routes of post-exposure prophylaxis against rabies. Heal J Indian Assoc Prev Soc Med 2015;6:30–5. http://iapsmgc.org/index_pdf/170.pdf. Accessed 25 Aug 2020

[24] World Health Organization. Animal bites 2018. https://www.who.int/en/news-room/fact-sheets/detail/animal-bites (accessed 22 May 2021).

[25] Rothe K, Tsokos M, Handrick W (2015). Animal and human bite wounds. Dtsch Arztebl Int.

[26] Dodet B (2010). Report of the sixth AREB meeting, Manila, The Philippines, 10–12 November 2009. Vaccine.

[27] Tarantola A, Blanchi S, Cappelle J, Ly S, Chan M, In S (2018). Rabies postexposure prophylaxis noncompletion after dog bites: estimating the unseen to meet the needs of the underserved. Am J Epidemiol.

[28] Penjor K, Marquetoux N, Dorji C, Penjor K, Dorjee S, Dorjee C (2020). Evaluation of post-exposure prophylaxis practices to improve the cost-effectiveness of rabies control in human cases potentially exposed to rabies in southern Bhutan. BMC Infect Dis.

[29] Ang ZY, Cheah KY, Shakirah MS, Fun WH, Anis-Syakira J, Kong YL (2021). Malaysia’s health systems response to COVID-19. Int J Environ Res Public Health.

[30] BERNAMA—COVID-19: overcome fear of coming to hospitals, clinics n.d. https://www.bernama.com/en/general/news_covid-19.php?id=1842454 (accessed 8 Mar 2022).

[31] Saleem SM, Quansar R, Haq I, Salim Khan SM (2021). Imposing COVID-19 lockdown and reported dog bite cases: an experience from a tertiary antirabies center of North India. J Vet Behav.

[32] Shantavasinkul P, Tantawichien T, Wilde H, Sawangvaree A, Kumchat A, Ruksaket N (2010). Postexposure rabies prophylaxis completed in 1 week: preliminary study. Clin Infect Dis.

[33] Dewan Negara Malaysia. Jawapan-jawapan Lisan bagi pertanyaan-pertanyaan Dewan Negara 3 Disember 2001. 2001.

[34] Palamar MB, Peterson MN, Deperno CS, Correa MT (2013). Assessing rabies knowledge and perceptions among ethnic minorities in Greensboro. North Carolina J Wildl Manage.

[35] Chew KS, Tan TW, Ooi YT (2011). Influence of chinese cultural health beliefs among malaysian chinese in a suburban population: a survey. Singapore Med J.

[36] Abdullah NH. Kenyataan Akhbar KKM 1 Julai 2017 – Kes Rabies di Negeri Sarawak – From the Desk of the Director-General of Health Malaysia 2017:3–7. https://kpkesihatan.com/2017/07/01/kenyataan-akhbar-kkm-1-julai-2017-kes-rabies-di-negeri-sarawak/ (accessed 4 May 2021).

[37] Abdullah NH. Kenyataan Akhbar KPK 1 Jun 2018 – Kes Terbaru Rabies di Kalangan Manusia di Sarawak – From the Desk of the Director-General of Health Malaysia 2018. https://kpkesihatan.com/2018/06/01/kenyataan-akhbar-kpk-1-jun-2018-kes-terbaru-rabies-di-kalangan-manusia-di-sarawak/ (accessed 13 June 2021).

[38] Abdullah NH. Kenyataan Akhbar KPK 13 Jun 2019 – Situasi Terkini Rabies di Sarawak – From the Desk of the Director-General of Health Malaysia 2019. https://kpkesihatan.com/2019/06/13/kenyataan-akhbar-kpk-13-jun-2019-situasi-terkini-rabies-di-sarawak/ (accessed 13 June 2021).

[39] Abdullah NH. Kenyataan Akhbar KPK 26 Jun 2020 – Situasi Terkini Rabies Di Sarawak – From the Desk of the Director-General of Health Malaysia 2020. https://kpkesihatan.com/2020/06/26/kenyataan-akhbar-kpk-26-jun-2020-situasi-terkini-rabies-di-sarawak/ (accessed 13 June 2021).

[40] Shrestha A, Rimal J (2018). Effectiveness of a mass media campaign on oral carcinogens and their effects on the oral cavity. ASian Pac J Cancer Prev.

[41] Gautret P, Labreuil C, Seyni M, Delmont J, Parola P, Brouqui P (2011). Effect of media warnings on rabies postexposure prophylaxis. France Emerg Infect Dis.

[42] Gadapani B, Rahini S, Manapurath R (2019). Noncompliance of the postexposure prophylactic vaccination following animal bites reporting to a rural primary health center. J Family Med Prim Care.

